# Medium-Term Effectiveness of a Comprehensive Internet-Based and Patient-Specific Telerehabilitation Program With Text Messaging Support for Cardiac Patients: Randomized Controlled Trial

**DOI:** 10.2196/jmir.4799

**Published:** 2015-07-23

**Authors:** Ines Frederix, Dominique Hansen, Karin Coninx, Pieter Vandervoort, Dominique Vandijck, Niel Hens, Emeline Van Craenenbroeck, Niels Van Driessche, Paul Dendale

**Affiliations:** ^1^ Mobile Health Institute Faculty of Medicine & Life Sciences Hasselt University Diepenbeek Belgium; ^2^ Cardiology Department of Cardiology Jessa Hospital Hasselt Belgium; ^3^ Expertise Center for Digital Media Faculty of Sciences Hasselt University Hasselt Belgium; ^4^ Cardiology Department of Cardiology Hospital East-Limburg Genk Belgium; ^5^ Patient Safety & Health Economics Institute Department of Patient Safety & Health Economics Hasselt University Diepenbeek Belgium; ^6^ Medicine and Health Sciences Institution Department of Public Health Ghent University Ghent Belgium; ^7^ Interuniversity Institute of Biostatistics and statistical Bioinformatics Interuniversity Institute of Biostatistics and statistical Bioinformatics Hasselt University Diepenbeek Belgium; ^8^ Vaccine and Infectious Disease Institute Center for Health Economic Research and Modelling Infectious Diseases University of Antwerp Antwerp Belgium; ^9^ Cardiology Department of Cardiology Antwerp University Hospital Antwerp Belgium

**Keywords:** telemedicine, eHealth, effectiveness, Internet

## Abstract

**Background:**

Cardiac telerehabilitation has been introduced as an adjunct or alternative to conventional center-based cardiac rehabilitation to increase its long-term effectiveness. However, before large-scale implementation and reimbursement in current health care systems is possible, well-designed studies on the effectiveness of this new additional treatment strategy are needed.

**Objective:**

The aim of this trial was to assess the medium-term effectiveness of an Internet-based, comprehensive, and patient-tailored telerehabilitation program with short message service (SMS) texting support for cardiac patients.

**Methods:**

This multicenter randomized controlled trial consisted of 140 cardiac rehabilitation patients randomized (1:1) to a 24-week telerehabilitation program in combination with conventional cardiac rehabilitation (intervention group; n=70) or to conventional cardiac rehabilitation alone (control group; n=70). In the telerehabilitation program, initiated 6 weeks after the start of ambulatory rehabilitation, patients were stimulated to increase physical activity levels. Based on registered activity data, they received semiautomatic telecoaching via email and SMS text message encouraging them to gradually achieve predefined exercise training goals. Patient-specific dietary and/or smoking cessation advice was also provided as part of the telecoaching. The primary endpoint was peak aerobic capacity (VO_2_ peak). Secondary endpoints included accelerometer-recorded daily step counts, self-assessed physical activities by International Physical Activity Questionnaire (IPAQ), and health-related quality of life (HRQL) assessed by the HeartQol questionnaire at baseline and at 6 and 24 weeks.

**Results:**

Mean VO_2_ peak increased significantly in intervention group patients (n=69) from baseline (mean 22.46, SD 0.78 mL/[min*kg]) to 24 weeks (mean 24.46, SD 1.00 mL/[min*kg], *P*<.01) versus control group patients (n=70), who did not change significantly (baseline: mean 22.72, SD 0.74 mL/[min*kg]; 24 weeks: mean 22.15, SD 0.77 mL/[min*kg], *P=*.09). Between-group analysis of aerobic capacity confirmed a significant difference between the intervention group and control group in favor of the intervention group (*P*<.001). At 24 weeks, self-reported physical activity improved more in the intervention group compared to the control group (*P=*.01) as did the global HRQL score (*P=*.01).

**Conclusions:**

This study showed that an additional 6-month patient-specific, comprehensive telerehabilitation program can lead to a bigger improvement in both physical fitness (VO_2_ peak) and associated HRQL compared to center-based cardiac rehabilitation alone. These results are supportive in view of possible future implementation in standard cardiac care.

## Introduction

Cardiovascular disease (CVD) causes more than 4 million deaths in Europe and approximately 2 million deaths in the European Union each year, attributing to 47% and 40% of all deaths, respectively [1]. After an acute cardiovascular event, the European Society of Cardiology (ESC) guidelines recommend cardiac rehabilitation to prevent recurrent disease for both coronary artery disease (CAD) [2] and chronic heart failure (CHF) [3] patients (Class IB). However, the long-term benefits of conventional center-based cardiac rehabilitation are often disappointing because of lack of adherence to prescribed lifestyle behavior [4]. Therefore, it is important to examine and introduce adjunct intervention strategies to stimulate adherence to a healthy lifestyle.

During the past years, cardiac telerehabilitation was introduced as an adjunct or alternative to conventional cardiac rehabilitation to increase uptake rates, enable more prolonged care, and improve long-term success. Two recent systematic reviews concluded telerehabilitation to be noninferior and/or superior when compared to standard cardiac rehabilitation [5,6]. However, the European Heart Network emphasizes the need for more studies to be carried out on eHealth interventions to ensure its effectiveness and cost-effectiveness before large-scale implementation in current health care systems [7].

The aim of this multicenter, prospective randomized controlled trial was to assess medium-term effectiveness of a patient-specific, comprehensive cardiac telerehabilitation program in addition to standard ambulatory cardiac rehabilitation. Contrary to most prior clinical trials on cardiac telerehabilitation, it included both telemonitoring and telecoaching strategies and focused on multiple cardiac rehabilitation core components (physical activity, nutritional counseling, and smoking cessation) [5]. It was hypothesized that the addition of cardiac telerehabilitation to standard cardiac rehabilitation leads to significant greater increments in physical activity level and physical fitness. This paper reports on the main study results.

## Methods

### Patient Recruitment

Telerehab III (ISRCTN29243064) was a multicenter, prospective randomized controlled clinical trial run at Jessa Hospital (Hasselt) (n=103), Ziekenhuis-Oost Limburg (Genk) (n=27), and St Franciscus Hospital (Heusden-Zolder) (n=10) in Belgium between February 2013 and 2015. Patients were recruited/enrolled over a timeframe of 19 months (from February 2013 to August 2014). A detailed description of the study protocol has been published previously [8].

Patients were eligible for participation in Telerehab III when they entered cardiac rehabilitation for (1) CAD and treated conservatively with a percutaneous coronary intervention or with coronary artery bypass grafting, (2) CHF with reduced ejection fraction (EF; New York Heart Association [NYHA] classes I, II, and III), or (3) CHF with preserved EF (NYHA I, II, and III as defined in the ESC guidelines). Patients were required to have a computer at home with Internet access (they had to be computer and Internet literate). The main exclusion criteria were (1) CHF NYHA class IV, (2) symptomatic and/or exercise-induced cardiac arrhythmia within the previous 6 months, (3) physical disability related to musculoskeletal or neurological problems, and (4) severe cognitive impairment. All patients provided offline informed consent after the nature and possible consequences of the study were explained before study enrollment (see Multimedia Appendix 1 for the informed consent). Patients were recruited offline at the hospitals’ rehabilitation centers by face-to-face information sessions. They were randomly assigned (1:1) to Internet-based telerehabilitation in addition to center-based rehabilitation (intervention group) or center-based rehabilitation alone (control group). A central computerized randomization system, using block randomization, ascertained equal distribution of patients in the different recruiting hospitals for both treatment arms.

The study was conducted in accordance with the principles stated in the Declaration of Helsinki (reviewed version of 2008), local and national regulations. The study protocol was approved by Jessa Ethics Committee (reference number: B243201216043). The trial is reported in accordance with CONSORT-EHEALTH (see Multimedia Appendix 2 for the completed CONSORT-EHEALTH form V1.6).

### Study Intervention

#### Center-Based Cardiac Rehabilitation Program

Both groups participated in a 12-week conventional center-based cardiac rehabilitation program, including 45 pluridisciplinary rehabilitation sessions with at least 2 exercise training sessions per week [9]. Patients were instructed to exercise for 45 to 60 minutes per session at a target heart rate and/or workload corresponding to an intensity between their first ventilatory threshold (VT_1_, as detected by V-slope method) and respiratory compensation point (RCP, as detected by carbon dioxide equivalent [VE/VCO_2_] slope method). Endurance training consisted of walking/running and/or cycling and arm cranking. They also had at least one consultation with the dietician and the psychologist of the rehabilitation center. The dietician provided the patients with general guidelines on healthy diet, the psychologist aimed to improve the patient’s self-efficacy to change prior unhealthy lifestyle behavior to a more healthy lifestyle behavior. He also assessed the patients’ potential mood disorders (eg, depression, anxiety) related to their cardiac event.

#### Telerehabilitation Program

Intervention group patients received a 24-week, Internet-based, comprehensive telerehabilitation program in addition to the conventional center-based cardiac rehabilitation. The telerehabilitation program started at week 6 of the 12-week center-based cardiac rehabilitation allowing the intervention group patients to become familiarized with the telerehabilitation’s motion sensor (Yorbody accelerometer, Belgium) and associated password-protected webservice during the 6-week overlap period. The program focused on multiple cardiac rehabilitation core components and used both physical activity telemonitoring and dietary/smoking cessation/physical activity telecoaching strategies. For the telemonitoring part, intervention group patients were prescribed patient-specific exercise training protocols based on achieved peak aerobic capacity (VO_2_ peak) during initial maximal cardiopulmonary exercise testing and calculated body mass index (BMI) [8]. Intervention group patients were instructed to continuously wear the accelerometer and to regularly transmit their registered activity data to the telerehabilitation center’s local server. They were instructed to transmit their physical activity data at least once weekly, but preferably daily. Data were transmitted to the telerehabilitation center’s local server in a few minutes after starting transmission. These data enabled a semiautomatic telecoaching system to provide the patients with feedback via email and short message service (SMS) text messaging (once weekly), encouraging them to gradually achieve predefined exercise training goals (see Multimedia Appendix 3 for a screenshot of the website with an example SMS text message sent to the patient). In addition, patients received emails and/or SMS text messages (once weekly) with tailored dietary and smoking cessation recommendations. The dietary telecoaching program included a module for diabetes mellitus, arterial hypertension, obesity, and a healthy module. Cardiovascular risk factor profiling at entry of study determined which module(s) were prescribed for each patient. The smoking cessation telecoaching program included information on major risks associated with smoking, the health benefits of smoking cessation, and nicotine replacement therapy. It provided smokers with encouraging messages toward smoking cessation.

The content of the feedback messages differed from the content of the center-based cardiac rehabilitation program in that it changed over time based on how well the patient changed his prior lifestyle behavior. For example, the exercise training feedback was intended to encourage patients to achieve predefined patient-specific training goals. If a patient succeeded in getting closer toward these predefined goals, the feedback would encourage the patient to improve his/her training even more. If the patient’s exercise training deteriorated during the study period, the feedback aimed to get the patient back on track. One independent person was responsible for technical assistance in case of sensor/system failure (part-time). One care provider supervised sent emails and/or SMS text messages and he/she was responsible for consistency and correctness of the content of sent messages. He/she also intervened in case of serious abnormal registrations (part-time). Access to registered data by the care provider was password-protected. The care provider that supervised sent emails and/or SMS text messages was a staff member that had coached cardiac patients for more than 5 years during their conventional center-based cardiac rehabilitation program. During the training period, this care provider also received a specific course on how to detect and what to do in case of alarming signs/symptoms. During the whole study period, one cardiologist supervised the care provider and was available to answer questions and to assist the care provider if necessary.

### Outcome Measures

All outcome assessors were blinded to group allocation. The primary outcome measure was peak aerobic capacity (VO_2_ peak); measured during maximal cardiopulmonary exercise testing [10] with breath-by-breath gas exchange analysis at baseline and after 6 and 24 weeks (Jaeger MS-CPX). The cardiopulmonary exercise test was maximal in case of an achieved heart rate >85% of the maximal predicted heart rate, a respiratory gas exchange ratio (RER) >1.1, and/or a ventilatory reserve (VR; VR=peak minute ventilation/maximal voluntary ventilation [VE peak/MVV]) >80% [10]. The first ventilatory threshold (VT_1_) and the oxygen uptake efficiency slope (OUES) were used as surrogate markers for VO_2_ peak in case of submaximal cardiopulmonary exercise test. VT_1_ was defined by the V-slope method; OUES was calculated using the method of Baba et al [11]*.* Two independent investigators, blinded to treatment allocation, interpreted cardiopulmonary exercise test reports.

The first secondary outcome measure was daily physical activity [12], both registered by triaxial accelerometry (Yorbody sensor) and self-assessed by the patient. The accelerometer provided daily recordings of aerobic (defined as sustained activity at ≥60 steps/min for ≥10 minutes), regular (activity at <60 steps/min), and total (sum of aerobic and regular) steps. Self-reported physical activity was based on the offline International Physical Activity Questionnaire (IPAQ) questionnaire, completed at baseline and after 6 and 24 weeks. Metabolic equivalent task (MET) minutes were computed by multiplying predefined MET scores by the minutes of a specific activity performed to weigh each type of activity by its energy requirement (for the domain leisure time activity and for all domains together). The following MET scores were used: 3.3 METs for walking, 4.0 METs for moderate physical activity, and 8.0 METs for vigorous physical activity.

Hemoglobin A_1c_ (HbA_1c_), glycemic control, and lipid profile were assessed by blood sampling at study start and after 24 weeks study period.

The 14-item offline HeartQol questionnaire was used to assess health-related quality of life (HRQL) at study start and after 6 and 24 weeks [13]. Mean (SD) scores were calculated for both the physical (10-item) and emotional (4-item) subscale. The proportion of patients at the floor (*floor effect* defined as the lowest possible score on the questionnaire) and at the ceiling (*ceiling effect* defined as the best possible score) was determined to assess sensitivity to positive and negative changes in HRQL.

Qualitative feedback on the cardiac telerehabilitation system was obtained from intervention group patients by special offline feedback forms (see Multimedia Appendix 4 for an example of the feedback form used). Intervention group patients were requested to fill in these forms after study completion.

### Statistical Analysis

Data analysis was performed using SPSS version 22 (SPSS Inc, Chicago, IL, USA) according to the intention-to-treat principle by assigned treatment group. Nonparametric alternatives were used for parametric statistics in case assumptions for the latter were violated. The Shapiro-Wilk test was used to assess normality. Paired *t* tests (parametric) or Wilcoxon signed rank tests (nonparametric) were used for within-group analysis; independent *t* tests (parametric) or Mann-Whitney *U* tests (nonparametric) for between-group analysis. Repeated measures ANOVA (parametric) or Friedman’s ANOVA (nonparametric) compared multiple dependent means. Chi-square tests were used in case of categorical variables; Fisher’s exact tests were used when expected frequencies were small. Pearson’s (*r*) or Spearman’s (ρ) correlation coefficients were calculated to express relationships between variables (bivariate correlations). The significance level for tests was 2-sided α=.05. Effect sizes for the HeartQol questionnaire were reported using the standardized response mean methodology (standardized response mean=[A–B]/D), where *A* and *B* are the mean scores at time 2 and time 1, respectively, and *D* represents the score change standard deviation [13]. Sensitivity analysis of accelerometric activity measurements was performed to cope with incomplete activity registrations. Inclusion thresholds of 1000, 2000, 3000, 4000, and 5000 total daily steps or ≥7, ≥8, and ≥9 daily measurement hours were arbitrarily chosen because these represented reliable registrations. All available data were used; no data imputation was performed for missing values. A priori sample size calculation yielded 140 necessary patients to detect a 20% effect size of the primary outcome measure (VO_2_ peak) [14] between groups (intervention group vs control group) with a statistical power of 95% at a 2-sided type I error level of .05 and a dropout rate of 30%.

## Results

**Figure 1:**
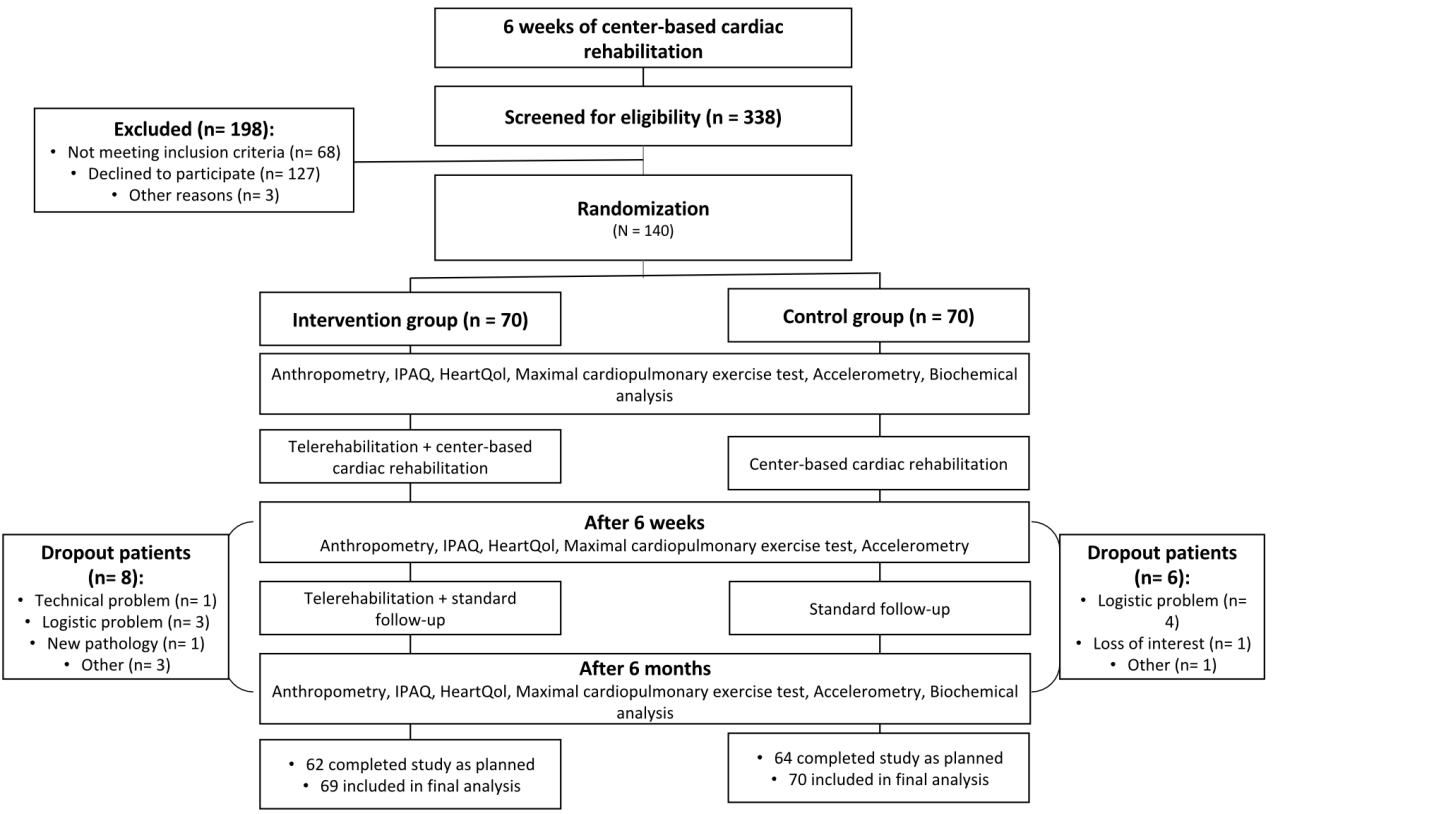
CONSORT patient flow diagram. IPAQ: International Physical Activity Questionnaire.

A total of 140 patients agreed to participate in the study, 70 patients in the control group and 70 patients in the intervention group (Figure 1). The numbers and reasons for dropout during study period were similar for both treatment groups. Dropout patients were included in the final analysis, with the exception of one intervention patient who was diagnosed with a noncardiac-related pathology (ie, lung cancer) and was excluded from final analysis. Intervention patients transmitted their activity data a mean 1.0 (SD 0.3) times per week. When averaged over the whole study period (24 weeks), 76% (52/69) of the intervention group patients did more than 2000 total daily steps or measured their physical activities 8 hours or more per day. Both treatment groups had similar baseline demographics, clinical characteristics, and medication use (Table 1).

**Table 1:** Baseline demographics, clinical characteristics, and medication use (N=140).

Characteristic			Intervention group (n=69)	Control group (n=70)	*P*
Age (years), mean (SD)			61 (9)	61 (8)	.95
**Gender, n (%)**					.38
	Female		10 (14)	15 (21)	
	Male		59 (96)	55 (79)	
**Type of cardiac pathology, n (%)**					.53
	Coronary artery disease		65 (94)	65 (93)	
	Heart failure with reduced ejection fraction		2 (3)	4 (6)	
	Heart failure with preserved ejection fraction		2 (3)	1 (1)	
**NYHA class,** ^a^ **n (%)**					.10
	I		54 (78)	61 (87)	
	II		12 (18)	4 (6)	
	III		3 (4)	5 (7)	
**Ejection fraction, n (%)**					.32
	>50%		52 (75)	50 (71)	
	35%-50%		0 (0)	3 (4)	
	<35%		17 (25)	17 (24)	
**Comorbidity, n (%)**					
	Atrial fibrillation		5 (7)	6 (9)	.99
	Diabetes mellitus		17 (25)	19 (27)	.85
	Hyperlipidemia		53 (77)	55 (79)	.84
	Arterial hypertension		40 (60)	44 (63)	.61
	Family history of cardiac disease		34 (49)	36 (51)	.87
	Peripheral artery disease		8 (12)	11 (16)	.62
**Smoking, n (%)**					.99
	Current smoker		18 (26)	18 (26)	
	Prior smoker		22 (32)	23 (33)	
	Nonsmoker		29 (42)	29 (41)	
Body mass index (kg/m^2^), mean (SD)			28 (5)	28 (4)	.54
**Medications, n (%)**					
	On beta blocker		53 (77)	57 (81)	.61
	On ACE-inhibitor		44 (64)	48 (69)	.72
	On statin		66 (96)	64 (91)	.16
	**On antiplatelet therapy**				.88
		Dual antiplatelet therapy	37 (54)	40 (57)	
		Antiplatelet monotherapy	29 (42)	27 (39)	
		No antiplatelet therapy	3 (4)	3 (4)	
	On diuretics		12 (17)	14 (20)	.76
	On oral antidiabetics		10 (15)	10 (14)	.94
	On insulin		7 (10)	5 (7)	.51
	On anticoagulative therapy		4 (6)	5 (7)	.76
	On antiarrhythmics		4 (6)	3 (4)	.67

^a^NYHA: New York Heart Association.

### Aerobic Capacity

**Figure 2:**
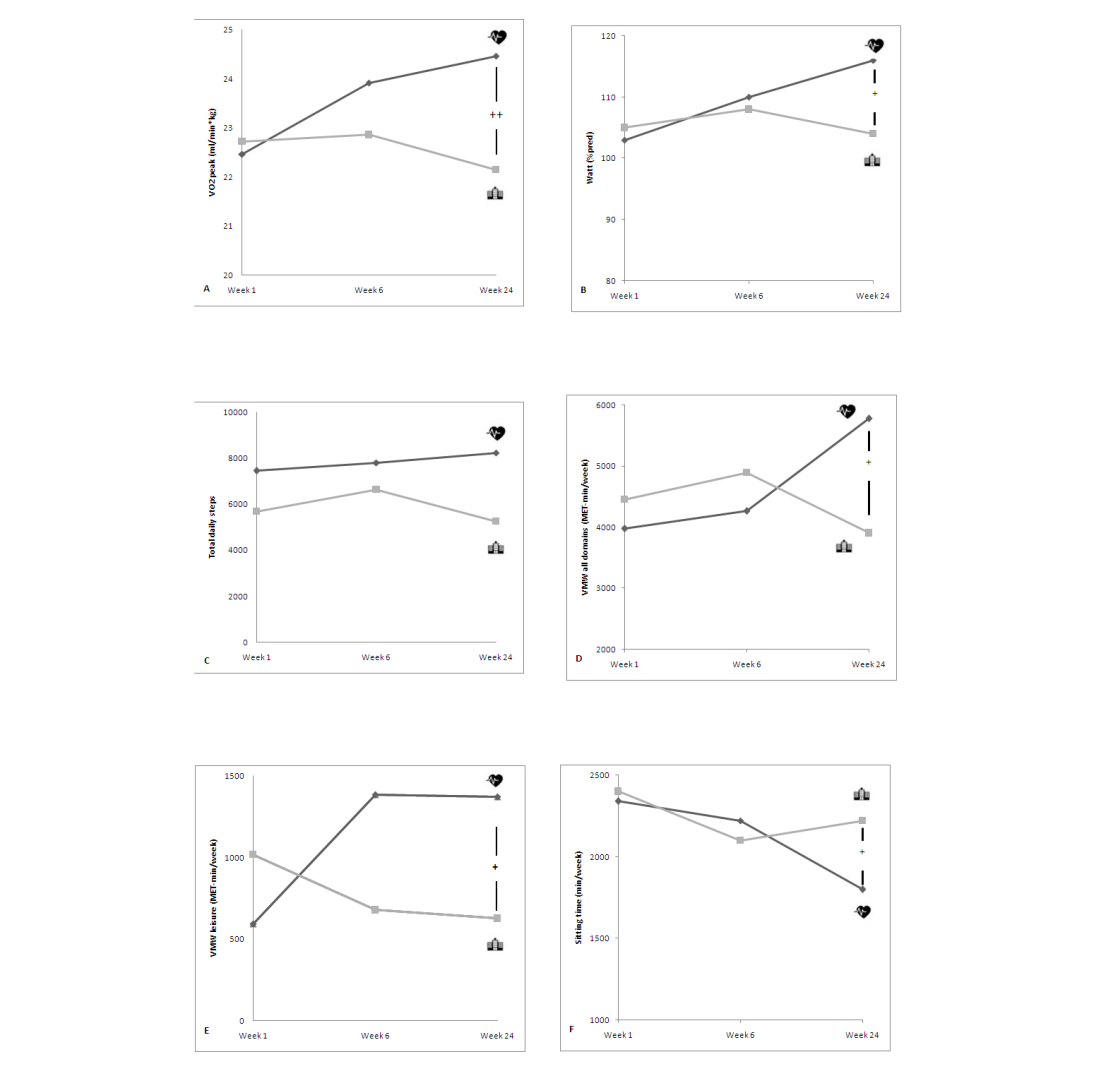
Line charts depicting (A) mean VO2 peak (mL/[min*kg]), (B) mean Watt (% predicted), (C) median total daily steps, (D) median vigorous-moderate-walking (VMW) activity for all domains (MET-min/week), (E) median VMW activity for leisure time (MET-min/week), (F) median sitting time (min/week) for week 1, week 6, and week 24, respectively. Intervention group is represented by the heart icon; control group by the building icon. *P<.05, ** P<.001.

Table 2 shows the cardiopulmonary exercise test outcome measures assessed at baseline and after 6 and 24 weeks. Mean VO_2_ peak improved significantly in intervention group patients from baseline (mean 22.46, SD 0.78 mL/[min*kg]) to 24 weeks (mean 24.46, SD 1.00 mL/[min*kg], *P*<.01). In the control group, mean VO_2_ peak did not change after 24 weeks when compared to baseline (*P=*.09) and decreased from week 6 (mean 22.86, SD 0.66 mL/[min*kg]) to week 24 (mean 22.15, SD 0.77 mL/[min*kg], *P*=.02) after an initial nonsignificant increase. Between-group analysis of aerobic capacity was significant after 24 weeks (*P*<.001) in favor for the intervention group. VT_1_ (Watt), OUES (mL/min/[log mL/min]), and Watt (% predicted) changed similarly over time (Figure 2) (see Multimedia Appendix 5 for a complete overview of the cardiopulmonary exercise test results).

**Table 2:** Cardiopulmonary exercise test parameters at baseline and 6 weeks and 24 weeks follow-up period.

Cardiopulmonary exercise test results^a^		Week, mean (SD)			Within-group, *P*				Between-group, *P*			
		Week 1	Week 6	Week 24	Overall	Δ 6-1	Δ 24-6	Δ 24-1	Overall	Δ 6-1	Δ 24-6	Δ 24-1
**Intervention group**												
	VO_2_ peak (mL/[min*kg])	22.46 (6.43)	23.91 (6.74)	24.46 (7.57)	.01	.08	.38	.01	<.001	.19	.01	<.001
	HR max (% pred)	79 (13)	80 (12)	83 (12)	.047	.99	.36	.05	.53	N/A	N/A	N/A
	Watt (W)	152 (48)	163 (52)	165 (53)	.01	.02	.81	.01	.01	.90	.01	.02
	Watt (pred%)	103 (23)	110 (27)	116 (27)	<.001	.01	.27	<.001	<.001	.83	.01	.01
	VT_1_ (W)	69 (24)	75 (25)	81 (26)	<.001	.74	<.001	<.001	<.001	.80	<.001	<.001
	VT_1_ (bpm)	93 (17)	91 (15)	96 (15)	.01	.99	.01	.08	.01	.35	.01	.047
	OUES (mL/min/log[mL/min])	2067 (518)	2241 (545)	2272 (579)	<.001	.02	.045	<.001	.1	N/A	N/A	N/A
	Weight (kg)	83.3 (18.2)	83.2 (17.4)	83.0 (17.3)	.69	N/A	N/A	N/A	.45	N/A	N/A	N/A
	BMI (kg/m^2^)	28 (5)	28 (5)	28 (5)	.63	N/A	N/A	N/A	.60	N/A	N/A	N/A
	DBP rest (mm Hg)	82 (19)	81 (21)	77.24 (21.13)	.48	N/A	N/A	N/A	.67	N/A	N/A	N/A
	SBP rest (mm Hg)	126 (21)	129 (30)	150 (140)	.26	N/A	N/A	N/A	.30	N/A	N/A	N/A
**Control group**												
	VO_2_ peak (mL/([min*kg])	22.72 (6.05)	22.86 (5.37)	22.15 (5.83)	.02	.99	.02	.09				
	HR max (% pred)	77 (12)	79 (13)	79 (12)	.43	N/A	N/A	N/A				
	Watt (W)	150 (49)	158 (50)	152 (53)	.01	<.001	.02	.99				
	Watt (pred%)	105 (26)	108 (26)	104 (27)	.01	<.001	.01	.99				
	VT_1_ (W)	83 (34)	88 (34)	76 (31)	<.001	.20	<.001	.01				
	VT_1_ (bpm)	95 (15)	96 (15)	95 (17)	.53	N/A	N/A	N/A				
	OUES (mL/min/log[mL/min])	2493 (2338)	2264 (637)	2142 (636)	.25	N/A	N/A	N/A				
	Weight (kg)	82.7 (13.4)	82.5 (13.3)	82.5 (13.9)	.18	N/A	N/A	N/A				
	BMI (kg/m^2^)	28 (4)	28 (4)	27 (5)	.51	N/A	N/A	N/A				
	DBP rest (mm Hg)	84 (21)	78 (19)	79 (17)	.33	N/A	N/A	N/A				
	SBP rest (mm Hg)	129 (25)	127 (23)	129 (21)	.57	N/A	N/A	N/A				

^a^ BMI: body mass index; CPET: cardiopulmonary exercise testing; DBP: diastolic blood pressure; HR: heart rate; N/A: not applicable; OUES: oxygen uptake efficiency slope; SBP: systolic blood pressure; VT_1_: first ventilatory threshold.

### Physical Activity

Sensitivity analysis of accelerometric step data confirmed similar activity patterns for both groups, regardless of the thresholds (See Multimedia Appendix 6 for sensitivity analysis). More than 2000 total daily steps or 8 or more daily measurement hours were used as thresholds for further analysis. In the intervention group, total daily steps increased from baseline (median 7448, IQR 24) to both 6 weeks (median 7799, IQR 37) and 24 weeks (median 8233, IQR 32); however, no changes were significant (*P*=.24). In the control group, total daily steps showed an initial increasing trend from baseline (median 5678, IQR 13) to week 6 (median 6630, IQR 11), but declined afterwards (median 5265, IQR 17, *P*=.85). Total daily steps were positively correlated with VO_2_ peak at baseline (ρ=.330, *P*=.01), 6 weeks (ρ=.237, *P*=.03), and 24 weeks (ρ=.485, *P*<.001).

Self-reported physical activity (by IPAQ questionnaire) was converted to MET-min/week of vigorous and/or moderate and/or walking (VMW) activities for the leisure time domain and all domains together, respectively. Summed leisure VMW increased significantly in the intervention group (based on Friedman's test, χ^2^_2_=13.7, *P=*.01) during the study period. In the control group, leisure VMW did not change (based on Friedman's test, χ^2^_2_=0.6, *P=*.72); however, it showed a downward trend (Figure 2). Between-group analysis confirmed a difference between the intervention group and control group in favor of the intervention group (*U*=1830, *z*=3.336, *P=*.01) (see Multimedia Appendix 7 for a summary of IPAQ questionnaire results). Contrary to the VMW activities, total sitting time decreased significantly during the study period in the intervention group (based on Friedman's test, χ^2^_2_=19.9, *P<*.001). In the control group, total siting time did not change overall (based on Friedman's test, χ^2^_2_=3.7, *P=*.16). Control patients tended to decrease sitting time during the first 6 weeks, but increased their sitting time back again after 6 weeks (Figure 1). Between-group analysis confirmed a difference between the intervention group and control group for self-reported total sitting time (*U*=1360, *z*=–2.427, *P*=.02).

### Cardiovascular Risk Factors

In the intervention group, no significant within-group differences were found for weight (*P=*.69), BMI (*P=*.63), diastolic blood pressure (*P=*.48), or systolic blood pressure (*P=*.26). The same was true for the control group (weight: *P=*.18; BMI: *P=*.51; diastolic blood pressure: *P=*.33; systolic blood pressure: *P=*.57). No between-group differences were found for these outcomes.

Fasting glucose levels, HbA_1c_, and LDL cholesterol did not change during the study period in the intervention group (*P=*.67, *P=*.18, and *P=*.20, respectively) nor in the control group (*P=*.25, *P=*.51, and *P=*.31, respectively). Total cholesterol levels increased in both treatment groups, but no between-group differences were found (*P=*.97).

### Health-Related Quality of Life

Intervention group patients showed a significant improvement in perceived HRQL for the physical subscale from baseline (mean 2.23, SD 0.08) to the end of study period (mean 2.52, SD 0.07; based on Friedman's test, χ^2^_2_=15.4, *P*<.001) (Table 3). The standardized response mean of 0.43 indicated a small to moderate effect size. Their global HRQL score also improved significantly (based on Friedman's test, χ^2^_2_=14.0, *P*<.001). The standardized response mean of 0.43 indicated a small to moderate effect size. The HRQL of the control group patients did not change during study period for the physical subscale (based on Friedman's test, χ^2^_2_=6.3, *P=*.05), the emotional subscale (based on Friedman's test, χ^2^_2_=0.5, *P=*.80), or the global scale (based on Friedman's test, χ^2^_2_=3.1, *P*=.21). Between-group analysis confirmed that globally the intervention group’s HRQL improved more than the control group (*U*=2407, *z*=2.805, *P=*.01).

### Qualitative Feedback

In all, 97% (67/69) of intervention group patients reported the telerehabilitation’s motion sensor was easy to read and 97% (67/69) found it easy to use. In general, patients were very satisfied (44%, 30/69) or satisfied (51%, 35/69) (total: 95%, 65/69 very satisfied/satisfied) with the telerehabilitation program. In the end, 89% (61/69) of patients were willing to use the system after study completion.

**Table 3:** Results from HeartQol questionnaire at baseline and after 6 weeks and 24 weeks of follow-up.

Score		Intervention group^a^					Control group^a^					*P*
		Baseline	Week 6	Week 24	*P*	SRM	Baseline	Week 6	Week 24	*P*	SRM	
**Physical subscale**												
	Mean (SD) score	2.23 (0.67)	2.45 (0.51)	2.52 (0.52)			2.27 (0.61)	2.39 (0.54)	2.28 (0.63)			
	Ceiling effect (%)	12%	14%	24%			11%	16%	17%			
	Floor effect (%)	0%	0%	0%			0%	0%	0%			
	Overall				<.001	.44				.05	N/A	.01
	Δ6-1				.046	.45				N/A	N/A	.05
	Δ24-6				.99	N/A				N/A	N/A	.03
	Δ24-1				<.001	.43				N/A	N/A	.01
**Emotional subscale**												
	Mean (SD) score	2.36 (0.75)	2.47 (0.70)	2.53 (0.54)			2.41 (0.70)	2.43 (0.65)	2.41 (0.69)			
	Ceiling effect (%)	30%	43%	34%			40%	35%	32%			
	Floor effect (%)	5%	2%	0%			0%	0%	0%			
	Overall				.14	N/A				.80	N/A	.22
	Δ6-1				N/A	N/A				N/A	N/A	N/A
	Δ24-6				N/A	N/A				N/A	N/A	N/A
	Δ24-1				N/A	N/A				N/A	N/A	N/A
**Global score**												
	Mean (SD) score	2.27 (0.63)	2.46 (0.51)	2.53 (0.44)			2.31 (0.59)	2.40 (0.51)	2.32 (0.58)			
	Ceiling effect (%)	8%	13%	19%			6%	10%	10%			
	Floor effect (%)	0%	0%	0%			0%	0%	0%			
	Overall				.01	.44				.21	N/A	.01
	Δ6-1				.07	.44				N/A	N/A	.05
	Δ24-6				.84	N/A				N/A	N/A	.04
	Δ24-1				<.001	.43				N/A	N/A	.01

^a^N/A: not applicable; SRM: standardized response mean.

## Discussion

This study showed that an additional 6-month, patient-specific, comprehensive telerehabilitation program can lead to a bigger improvement in both physical fitness (VO_2_ peak) and associated HRQL compared to center-based cardiac rehabilitation alone. The real difference between both groups occurred after center-based cardiac rehabilitation was completed. The VO_2_ peak, daily total step count, and IPAQ’s self-reported VMW activities increased from baseline to 6 weeks in both treatment groups. They additionally increased between weeks 6 and 24 in the intervention group, but decreased in the control group. Control group patients participated in the center-based cardiac rehabilitation during the first 6 weeks of the study period only, probably explaining their initial improvement in outcome measures. The observed findings imply that control group patients return to prior lifestyle behavior after center-based cardiac rehabilitation, whereas intervention group patients further maintain and ameliorate acquired behavioral change. The proportions of dropout patients in the recruiting hospitals (9%, 9/103 for Jessa Hospital; 7%, 2/27 for Ziekenhuis-Oost Limburg; and 30%, 3/10 for St. Franciscus Hospital) were relatively low compared with dropout rates in standard cardiac rehabilitation programs.

Recent literature findings confirmed telehealth interventions such as telemonitoring to be feasible and effective for heart failure patients [15-18]. Furthermore, 2 systematic reviews on cardiac teleinterventions were published [5,6] . We reported on cardiac telerehabilitation in CAD and CHF patients with a total of 13,248 patients enrolled in 37 studies and a mean follow-up period of 9 months. We concluded that telerehabilitation was associated with significantly lower lack of adherence to physical activity guidelines (OR 0.56, 95% CI 0.45-0.69) [19-27]. However, Huang et al [6] found no statistically significant difference between telehealth interventions and center-based cardiac rehabilitation for exercise capacity (standardized mean difference [SMD]=−0.01, 95% CI −0.12 to 0.10), weight (SMD -0.13, 95% CI -0.30 to 0.05), systolic and diastolic blood pressure (mean difference [MD] -1.27, 95% CI -3.67 to 1.13 and MD 1.00, 95% CI -0.42 to 2.43, respectively), and lipid profile. Another recent systematic review by Widmer et al [28] on digital health interventions concluded that digital health interventions can improve cardiovascular risk factors such as weight loss, blood pressure, and LDL cholesterol in patients seeking primary prevention of CVD. In contrast, they found no consistent reductions in the aforementioned risk factors in secondary prevention studies.

The somewhat contrary findings between the review of Frederix et al [5]*,* the results of the current Telerehab III trial (ISRCTN29243064) showing effectiveness of cardiac teleinterventions on exercise capacity, and the review of Huang et al [6] that showed no effect on exercise capacity, could be attributed to differences in intervention group programs. It appears that a comprehensive teleintervention, including at least physical activity telemonitoring and telecoaching, is necessary. The feedback provided by the teleintervention should be patient-specific to increase success rates.

In this Telerehab III trial, we found no significant effect of the additional cardiac telerehabilitation program on weight loss, blood pressure, lipid profile, and/or glycemic control. This is consistent with the findings of Huang et al [6] and Widmer et al [28]. Digital health interventions seem to be able to improve cardiovascular risk factors in primary prevention, but not secondary prevention programs. Future research should focus on furthering our understanding of the variables determining this success of digital health interventions in primary prevention populations, contrary to secondary prevention populations.

The intervention group patients could see and follow up their own transmitted activity data by logging onto the Telerehab III webpage as many times as they preferred. On average, they transmitted their activity data and logged onto the webpage a mean 1.0 (SD 0.3) times per week. For some patients, their frequency of data transmission increased during the study period; the frequency of others remained stable. There were almost no patients for whom the frequency of data transmission decreased during study period.

The reason for the increasing frequency of data transmission, seen for some of the intervention patients, remains unclear. In this trial, all intervention patients received feedback messages with the same frequency (once weekly). However, it would be interesting to investigate if the patients’ frequency of data transmission would be different for different frequencies of sent feedback messages.

The strength of Telerehab III is that it, contrary to most analyzed trials in the review of Huang et al [6], provided intervention group patients with a comprehensive, patient-specific telerehabilitation program focusing on multiple core components (exercise training, nutritional counseling, smoking cessation). Both telecoaching and telemonitoring strategies were included; exercise training programs and dietary prescriptions were based on initial maximal cardiopulmonary exercise testing, BMI, and individual cardiovascular risk factor profile.

A limitation of this study was that Telerehab III was initially designed to recruit a broad cardiac patient population (including both CAD, CHF with reduced EF, and CHF with preserved EF). However, as shown by the baseline clinical characteristics (Table 1), only a minority of CHF patients eventually participated (5.8% and 7.1% in the intervention and control groups, respectively). This reduced the generalizability of study findings for CHF patients.

Sensitivity analysis of accelerometric activity measurements, based on arbitrarily selected thresholds, was performed to cope with incomplete activity registrations. Although the analysis found similar activity patterns for both groups regardless of chosen thresholds, it remains unclear whether the finally used thresholds of more than 2000 total daily steps or 8 or more daily measurement hours were most representative of reality. Therefore, one needs to interpret the accelerometric measurements with caution.

Finally, in Telerehab III, one part-time person (caregiver) was responsible for control of feedback content and one part-time person (technical assistant) for system/service operability. In a routine application setting, similar staff requirements would be sufficient.

This paper shows the addition of the cardiac telerehabilitation program to conventional center-based cardiac rehabilitation to be more effective than center-based cardiac rehabilitation alone in improving VO_2_ peak, self-reported physical activity, and associated HRQL at 24 weeks. We plan to conduct a follow-up trial of Telerehab III (starting in August 2015) to assess whether the intervention-related health benefits persist 2 years after study termination. The current findings answer to the European Heart Network’s question to profoundly well-document and evaluate critical eHealth interventions before large-scale deployment in the health care system. Future research should focus on even more elaborate comprehensive telerehabilitation programs that have the potential to improve not only aerobic capacity, physical activity level, and quality of life, but also improve the patient’s cardiovascular risk factor profile (weight, blood pressure, lipids, and glycemia control).

## Appendix

Multimedia Appendix 1 Informed consent.

Multimedia Appendix 2 CONSORT-EHEALTH checklist V1.6.2 [29].

Multimedia Appendix 3 Screenshot of the website with an example SMS sent to the patient.

Multimedia Appendix 4 Feedback form for Telerehab III intervention group.

Multimedia Appendix 5 Complete set of measured cardiopulmonary exercise test parameters at baseline, 6 weeks and 24 weeks follow-up period.

Multimedia Appendix 6 A Sensitivity analysis step data. B Use of telemonitoring system.

Multimedia Appendix 7 Results from IPAQ questionnaire at baseline, 6 weeks and 24 weeks of follow-up period.

## Abbreviations

BMI: body mass index
CAD: coronary artery disease
CHF: chronic heart failure
EF: ejection fraction
ESC: European Society of Cardiology
HRQL: health-related quality of life
IPAQ: International Physical Activity Questionnaire
MET: metabolic equivalent task
NYHA: New York Heart Association
OUES: oxygen uptake efficiency slope
RCP: respiratory compensation point
SMS: short message service
VMW: vigorous-moderate-walking
VO2 peak: peak aerobic capacity
