# Supplementary material for: Medium-Term Effectiveness of a Comprehensive Internet-Based and Patient-Specific Telerehabilitation Program With Text Messaging Support for Cardiac Patients: Randomized Controlled Trial
Source: J Med Internet Res. 2015 Jul 23;17(7):e185. doi: 10.2196/jmir.4799 (PMC4528085; doi:10.2196/jmir.4799)
Supplement: Multimedia Appendix 1 [file jmir_v17i7e185_app1.pdf]

Title of the study: Telerehab III<br>Medical Ethics Committee: Jessa Ethics Committee, Stadsomvaart 11, 3500 Hasselt<br>Local investigators: M.D. Ines Frederix, M.D. PhD Paul Dendale

I Information vital to your decision to take part

Introduction

You are being invited to take part in a clinical study.

Before you agree to take part in this study, we invite you to take note of its implications in terms of organisation, possible risks and benefits, to allow you to make a decision with full awareness of the implications. This is known as giving “informed consent”.

Please read these few pages of information carefully and ask any questions you want to the investigator or his/her representative.

There are 3 parts to this document: the information essential to your decision, your written consent and supplementary information (appendices) detailing certain aspects of the basic information.

If you take part in this study, you should be aware that:

- This clinical study is being conducted after having been reviewed by one or more ethics committees.
- Your participation is voluntary and must remain free from any coercion. It requires the signature of a document expressing your consent. Even after having signed this document, you can stop taking part by informing the investigator.
- The data collected on this occasion are confidential and your anonymity is guaranteed during publication of the results.
- Insurance has been taken out in case you should suffer any damage in connection with your participation in this clinical study.
- You may contact the investigator or a member of his/her team at any time should you need any additional information.

Objectives and course of the study

This clinical study has been organised to assess long-term effectiveness of a patient-specific, comprehensive cardiac telerehabilitation program in addition to standard ambulatory cardiac rehabilitation.

This clinical study is to include 140 patients, all in Belgium.

To be able to take part in this study, following inclusion and exclusion criteria were defined:

Inclusion criteria

ia. CAD patients treated conservatively, with PCI or CABG

ib. CHF patients with reduced EF

ic. CHF patients with preserved EF

ii. Current active rehabilitation in one of the recruiting centres

iii. Possesion of personal computer with internet connection

iv. Age > 18 and < 80 years

v. Familial with Dutch language

vi. Informed consent

Exclusion criteria

i. Orthopaedic and/or neurological condition, limiting the patient’s ability to actively engage in exercise training sessions

ii. Impairment to use the telerehabilitation equipment or appear at follow-up visits (terminal disease, dementia and cognitive impairment)

iii. Simultaneous participation in anoter clinical trial

iv. Patients with CHF NYHA IV

v. Patients with a history of VF, exertional sustained VT/supraventricular tachycardia within the previous 6 months

Patients in the control group receive standard cardiac rehabilitation (12-week program) in ReGo. Intervention patients receive standard cardiac rehabilitation plus telerehabilitation. For the telerehabilitation you will receive a motion sensor to monitor daily physical activities. Based on uploaded data, you will receive feedback. Also, you will receive telecoaching via email/SMS in order to adapt healthy diet (patient-specific dietary advices will be given) and to stop smoking (in case of smoker).

<u>Description of risks and benefits</u>

Although very small, there could be some risk, in terms of health, linked to the exercise training program (as part of the cardiac telerehabilitation program). Study insurances are provided.

Similarly, you should not expect any personal benefits as a result of taking part in the study. Know only that your participation will allow us to better understand the long-term effectiveness of a patient-specific, comprehensive cardiac telerehabilitation program in addition to standard ambulatory cardiac rehabilitation to offer better treatments in the future.

<u>Withdrawal of consent</u>

Your participation is voluntary and you are entitled to withdraw your consent to take part in the study for any reason, without having to justify your decision.

<u>If you take part in this study, we ask you:</u>

- To cooperate fully in the smooth running of this study.
- Not to conceal anything such as information relating to your state of health, the medication you are taking or the symptoms you are experiencing.
- To inform your doctor if you are asked to take part in another study to discuss with him/her the possibility of taking part in this study and to see whether you should then stop taking part in the present study.

Contact

If you need further information, but also if you have problems or concerns, you can contact the investigator (Frederix, Ines) on the following telephone number +3216583382.

If you have any questions relating to your rights as a participant in a clinical study, you can contact the patient rights ombudsman of your institution on this telephone number: +3211 30 84 00. If necessary, he/she can put you in contact with the ethics committee.

Title of the study: Telerehab III

II Informed consent

Participant

I declare that I have been informed of the nature of the study, its purpose, its duration, the possible side effects and what is expected of me. I have taken note of the information document and the appendices to this document.

I have had sufficient time to think about it and discuss it with a person of my choice (GP, relative).

I have had the opportunity to ask any questions that came to mind and have obtained a favourable response to my questions.

I understand that data about me will be collected throughout my participation in this study and that the investigator and the sponsor of the study will guarantee the confidentiality of these data.

I agree to my personal data being processed as described in the section dealing with confidentiality guarantees. I also consent to these data being transferred to and processed in countries other than Belgium.

I have received a copy of the information to the participant and the informed consent form.

Surname, first name, date and signature of the volunteer.

Investigator

I, the undersigned, Frederix Ines investigator, confirm that I have verbally provided the necessary information about the study and have given the participant a copy of the information document.

I confirm that no pressure was applied to persuade the patient to agree to take part in the study and that I am willing to answer any additional questions if required.

I confirm that I operate in accordance with the ethical principles set out in the latest version of the “Helsinki Declaration”, the “Good Clinical Practices” and the Belgian Law of 7 May 2004 related to experiments on humans.

Surname, first name, date and signature of the investigator’s representative

Surname, first name, date and signature of the investigator

III Supplementary information

1: Supplementary information on the protection and rights of the participant in a clinical study

Ethics Committee

This study has been reviewed by an independent Ethics Committee, namely the Ethics Committee of Jessa, which has issued a favourable opinion after consulting with the Ethics Committees of each centre where this trial will be conducted. It is the task of the Ethics Committees to protect people who take part in a clinical trial. They make sure that your rights as a patient and as a participant in a clinical study are respected, that based on current knowledge, the study is scientifically relevant and ethical.
You should not under any circumstances take the favourable opinion of the Ethics Committee as an incentive to take part in this study.

Voluntary participation

Before signing, do not hesitate to ask any questions you feel are appropriate. Take the time to discuss matters with a trusted person if you so wish.

Your participation in the study is voluntary and must remain free of any coercion: this means that you have the right not to take part in the study or to withdraw without giving a reason, even if you previously agreed to take part. Your decision will not affect your relationship with the investigator or the quality of your future therapeutic care.

If you agree to take part in this study, you will sign the informed consent form. The investigator will also sign this form to confirm that he/she has provided you with the necessary information about the study. You will receive a copy of the form.

Costs associated with your participation

The sponsor has arranged to compensate the hospital for the time devoted to the study by the investigator and his/her team. You will not receive any compensation for your participation in this study. Furthermore, the study will not involve any additional costs for you.

Guarantee of confidentiality

Your participation in the study means that you agree to the investigator collecting data about you and to the study sponsor using these data for research purposes and in connection with scientific and medical publications.

The investigator has a duty of confidentiality vis-à-vis the data collected.

This means that he/she undertakes not only never to reveal your name in the context of a publication or conference but also that he/she will encode your data before sending them to the manager of the database of collected data.

The investigator and his/her team will therefore be the only ones to be able to establish a link between the data transmitted throughout the study and your medical records.

The personal data transmitted will not contain any combination of elements that might despite everything allow you to be identified.

To verify the quality of the study, it is possible that your medical records will be examined by third parties (ethics committee, representatives of the study sponsor, external auditors). In any event, this may only take place under the responsibility of the investigator or of one of his/her colleagues and by persons subject to the obligation of professional secrecy.

These (encoded) data will be able to be sent to Belgian or other regulatory authorities, to the relevant ethics committees, to other doctors and/or to organisations working in collaboration with the sponsor.

Your consent to take part in this study therefore also implies your consent to the use of your encoded medical data for the purposes described in this information form and to their transmission to the aforementioned people and authorities.

Insurance

Even without fault, the sponsor accepts responsibility for damage caused to the participant (or his/her dependants) and linked directly or indirectly to participation in this study. In this context, the sponsor has taken out an insurance contract.
