# Supplementary material for: Medium-Term Effectiveness of a Comprehensive Internet-Based and Patient-Specific Telerehabilitation Program With Text Messaging Support for Cardiac Patients: Randomized Controlled Trial
Source: J Med Internet Res. 2015 Jul 23;17(7):e185. doi: 10.2196/jmir.4799 (PMC4528085; doi:10.2196/jmir.4799)
Supplement: Multimedia Appendix 2 [file jmir_v17i7e185_app2.pdf]

CONSORT-EHEALTH Checklist V1.6.2 Report

(based on CONSORT-EHEALTH V1.6), available at [http://tinyurl.com/consort-ehealth-v1-6].

4799

Date completed

5/31/2015 13:04:55

by

Ines

Long-term effectiveness of an internet-based, comprehensive and patient-tailored telerehabilitation program with short message service support for cardiac patients: Randomized Controlled Trial

TITLE

1a-i) Identify the mode of delivery in the title
The title includes the requested information.
The title is "Long-term effectiveness of an internet-based, comprehensive and patient-tailored telerehabilitation program with short message service support for cardiac patients: Randomized Controlled Trial."

1a-ii) Non-web-based components or important co-interventions in title
The title includes the requested information.
The title is "Long-term effectiveness of an internet-based, comprehensive and patient-tailored telerehabilitation program with short message service support for cardiac patients: Randomized Controlled Trial."

1a-iii) Primary condition or target group in the title
The title includes the requested information.
The title is "Long-term effectiveness of an internet-based, comprehensive and patient-tailored telerehabilitation program with short message service support for cardiac patients: Randomized Controlled Trial."

ABSTRACT

1b-i) Key features/functionalities/components of the intervention and comparator in the METHODS section of the ABSTRACT
The paper does not explicitly mention subitem 1b-i.
However, the design and development of the webservice and/or email/SMS service was based on the behavioural theory of Prochaska and DiClemente. In this model the feedback to the patient is tailored based on the patient's phase of behavioural change.

1b-ii) Level of human involvement in the METHODS section of the ABSTRACT
Subitem 1b-ii was addressed in the abstract as follows: "Based on registered activity data, they received semi-automatic telecoaching via e-mail and SMS, encouraging them to gradually achieve predefined exercise training goals. "

1b-iii) Open vs. closed, web-based (self-assessment) vs. face-to-face assessments in the METHODS section of the ABSTRACT
Subitem 1b-iii was addressed in the abstract as follows: "The primary endpoint was peak aerobic capacity (VO2peak). Secondary endpoints included accelerometer recorded daily step counts, self-assessed physical activities by IPAQ and quality of life (QoL), assessed by the HeartQol questionnaire at baseline, 6 weeks and 24 weeks study period."

1b-iv) RESULTS section in abstract must contain use data
Subitem 1b-iv is addressed in the abstract as follows: "Mean VO2peak increased significantly in IG patients (n = 69) from baseline [22.46 ± 0.78 ml/kg/min] to 24 weeks [24.46 ± 1.00ml/kg/min] (p < 0.001), contrary to CG patients (n = 70) where it did not change significantly (from 22.72 ± 0.74 ml/kg/min to 22.15 ± 0.77 ml/kg/min, p = 0.089). At 24 weeks, self-reported physical activity (MET-min/week) improved more in the IG, when compared to the CG (p = 0.001), as did the global QoL score (p = 0.005)."

1b-v) CONCLUSIONS/DISCUSSION in abstract for negative trials
The abstract of the artcile has a conclusion, namely: "This paper showed the addition of cardiac telerehabilitation to conventional center-based CR to be more effective than center-based CR alone in increasing aerobic capacity, physical activity level and associated QoL. These results are supportive in view of possible future implementation in standard cardiac care."

The trial was not negative.

INTRODUCTION

2a-i) Problem and the type of system/solution
The problem is stated in the article as follows: ". After an acute cardiovascular event, the European Society of Cardiology (ESC) guidelines recommend cardiac rehabilitation (CR) to prevent recurrent disease for both coronary artery disease (CAD)2 and chronic heart failure (CHF)3 patients (Class IB). The long-term benefits of conventional center-based CR are often disappointing however, due to lack of adherence to prescribed life style behavior4. It is therefore important to examine and introduce adjunct intervention strategies to stimulate adherence to a healthy lifestyle.
During the last decade, cardiac telerehabilitation was introduced as an adjunct or alternative to conventional CR in order to increase uptake rates, to enable more prolonged care and to improve long-term success. "
The intended particular patient population is a cardiac patient population (coronary artery disease and heart failure patients).

The goal of the intervention (in addition to the standard cardiac rehabilitation program) is to be more (cost-)effective than standard cardiac rehabilitation alone.

2a-ii) Scientific background, rationale: What is known about the (type of) system
What is known about telerehabilitation for cardiac patients? This is stated in the article as:

"Two systematic reviews on cardiac tele-interventions were published recently5,6 . Frederix et al. reported on cardiac telerehabilitation in CAD and CHF patients with a total of 13,248 patients enrolled in 37 studies; and a mean follow-up period of 9 months. They concluded that telerehabilitation was associated with significantly higher adherence rates to physical activity guidelines (Odds Ratio (OR) = 0.56, 95 % CI: 0.45-0.69)15-23. Huang et al. however, found no statistically significant difference between telehealth interventions and center-based CR for exercise capacity (standardized mean difference (SMD) = −0.01; 95 % CI: −0.12-0.10), quality of life and psychosocial state. The difference between these results and our findings could be attributed to differences in IG programs."

So there is evidence on the effectiveness of cardiac telerehabilitation. Contrary to prior studies however, Telerehab III is comprehensive (it includes both telemonitoring and telecoaching) and it focuses on multiple core components of cardiac rehabilitation (physical activity, diet, smoking cessation).

It is logical to compare the new intervention (telerehabilitation) to the current gold standard (standard cardiac rehabilitation i.e. the comparator).

METHODS

3a) CONSORT: Description of trial design (such as parallel, factorial) including allocation ratio

Subitem 2b is addressed as follows in the introduction:
"The aim of this multi-center, prospective randomized, controlled trial was to assess long-term effectiveness of a patient-specific, comprehensive cardiac telerehabilitation program in addition to standard ambulatory CR. Contrary to most prior clinical trials on cardiac telerehabilitation, it included both telemonitoring and telecoaching strategies and focused on multiple CR core components (physical activity, nutritional counselling and smoking cessation). It was hypothesized that the addition of cardiac telerehabilitation to standard CR leads to significant greater increments in physical activity level and physical fitness. This paper reports on the main study results."

3b) CONSORT: Important changes to methods after trial commencement (such as eligibility criteria), with reasons

There were no important changes to the methods after trial commencement.

3b-i) Bug fixes, Downtimes, Content Changes

There were no important changes made to the intervention or comparator during the trial (such as major bug fixes or changes in the functionality or content).
There were no important unexpected events that may have influenced study design such as staff changes, system failures/downtimes.

4a) CONSORT: Eligibility criteria for participants

The eligibility criteria for participants are present in the methods section as follows:
"Patients were eligible for participation in Telerehab III when they entered CR for (i) CAD and treated conservatively, with a percutaneous coronary intervention or with coronary artery bypass grafting; (ii) CHF with reduced EF (NYHA I, II and III) or (iii) CHF with preserved EF (NYHA I, II and III) (as defined in the ESC guidelines). Patients were required to have a computer at home with internet access (they had to be computer and internet literate). The main exclusion criteria were (i) CHF NYHA class IV, (ii) symptomatic and/or exercise induced cardiac arrhythmia within the previous six months, (iii) physical disability related to musculoskeletal or neurological problems and (iv) severe cognitive impairment. "

4a-i) Computer / Internet literacy

Computer/Internet literacy was an inclusion criterium as defined in the eligibility criteria as follows:
"Patients were required to have a computer at home with internet access (they had to be computer and internet literate). "

4a-ii) Open vs. closed, web-based vs. face-to-face assessments:

Subitem 4a-ii is addressed in the methods section as follows:

"Patients were recruited offline at the hospitals' rehabilitation centers by face-to-face information sessions."

4a-iii) Information giving during recruitment

Subitem 4a-iii is addressed in the methods section as follows:

"All patients provided offline informed consent, including a statement on the content of the intervention of interest, prior to study enrollment. "

The informed consent will be attached as appendix to the paper.

4b) CONSORT: Settings and locations where the data were collected

The self-assessed outcomes (self-reported physical activity by IPAQ questionnaire and self-reported quality of life by HeartQol questionnaire) were assessed through offline questionnaires.

Institutional affiliations were displayed to potentional participants by including the affiliations' logo's on flyers, the webpage, the questionnaires,...

4b-i) Report if outcomes were (self-)assessed through online questionnaires

The self-assessed outcomes (self-reported physical activity by IPAQ questionnaire and self-reported quality of life by HeartQol questionnaire) were assessed through offline questionnaires.

4b-ii) Report how institutional affiliations are displayed

Institutional affiliations were displayed to potentional participants by including the affiliations' logo's on flyers, the webpage, the questionnaires,...

5) CONSORT: Describe the interventions for each group with sufficient details to allow replication, including how and when they were actually administered

5-i) Mention names, credential, affiliations of the developers, sponsors, and owners

Subitem 5-i is addressed in the methods section as follows:

"Study intervention
1. Center-based CR program
Both groups participated in a 12-week conventional center-based CR program, including 45 pluridisciplinary rehabilitation sessions with at least two exercise training sessions per week10. Patients were instructed to exercise for 45-60 min per session at a target heart rate and/or workload corresponding to an intensity between their first ventilatory threshold (VT1, as detected by V-slope method) and respiratory compensation point (RCP, as detected by carbon dioxide equivalent (VE/VCO2) slope method). Endurance training consisted of walking/running, and/or cycling and arm cranking.
2. Telerehabilitation program
IG patients received a 24-week internet-based, comprehensive telerehabilitation program in addition to the conventional center-based CR. The telerehabilitation program started at week six of the center-based CR; allowing the IG patients to become familiarized with the telerehabilitation's motion sensor (Yorbody accelerometer) and associated password-protected webservice during the 6-week overlap period. The program focused on multiple CR core components and used both physical activity telemonitoring and dietary/smoking cessation/physical activity telecoaching strategies. For the telemonitoring part, IG patients were prescribed with patient-specific exercise training protocols, based on achieved peak aerobic capacity (VO2peak) during initial maximal cardiopulmonary exercise testing (CPET) and calculated body mass index (BMI) 8. IG patients were instructed to continuously wear the accelerometer and to weekly transmit their registered activity data to the telerehabilitation center's local server. These data enabled a semi-automatic telecoaching system to provide the patients with feedback via e-mail and SMS (once weekly), encouraging them to gradually achieve predefined exercise training goals. In addition patients received e-mails and/or SMS's (once weekly) with tailored dietary and smoking cessation recommendations, based on cardiovascular risk factors at entry of study. One independent person was responsible for technical assistance in case of sensor/system failure (part-time). One care provider supervised sent e-mails and/or SMS's, he/she was responsible for consistency and correctness of the content of sent messages. He/she also intervened in case of serious abnormal registrations (part-time). Access to registered data by the care provider was password-protected."

5-ii) Describe the history/development process

The telerehabilitation program applied in Telerehab III underwent extensive previous evaluation in the Telerehab II trial (including 80 patients). Telerehab II was a monocentric randomized controlled trial that assessed feasibility and usability issues. The program was adapted after Telerehab II, based on the results of this trial. As such, high adoption/use rates were expected in Telerehab III.

5-iii) Revisions and updating

Major intervention changes were made after Telerehab II and before Telerehab III.

During Telerehab III, the development and/or content was frozen.

5-iv) Quality assurance methods

Since the content of the feedback messages was developed by cardiologists, this content was considered of good quality.

The results of Telerehab II indicated good accuracy and quality of accelerometric registered physical activity data and/or the webservice that was consequently used in Telerehab III.

5-v) Ensure replicability by publishing the source code, and/or providing screenshots/screen-capture video, and/or providing flowcharts of the algorithms used

The flowchart of the algorithms used was described in detail elsewhere.

The reference where you can find this information is: Frederix I, Van Craenenbroeck EM, Vrints C, Dendale et al. Telerehab III: a multi-center randomized, controlled trial investigating the long-term effectiveness of a comprehensive cardiac telerehabilitation program - Rationale and study design. BMC Cardiovasc Disord 2015;15(1):29.

5-vi) Digital preservation

The URL of the application was removed after study termination.

5-vii) Access

Patients accessed the application with a specific password. They did not have to pay for the application.

Patients had to be included in the Telerehab III intervention group, in order to obtain access to the application.

5-viii) Mode of delivery, features/functionalities/components of the intervention and comparator, and the theoretical framework

Theoretical framework used: we based our intervention on the behavioural model of Prochaska and DiClemente. In this model feedback to the patient is tailored to the patient, based on his/her phase of behavioural change.

Description of the content: the content of the feedback messages (on physical activities, diet and smoking cessation) was developed by a team of cardiologists specialised in cardiac rehabilitation. It was designed in such a way that it encouraged the patient to achieve his/her predefined and personal rehabilitation goals.

Email and/or SMS were used as communication delivery channels.

5-ix) Describe use parameters

Patients in the intervention group wore the motion sensor all day long (7 days per week). They were instructed to upload and transmit measured data once weekly.

5-x) Clarify the level of human involvement

One independent person was responsible for technical assistance in case of sensor/system failure (part-time). One care provider supervised sent e-mails and/or SMS's, he/she was responsible for consistency and correctness of the content of sent messages. He/she also intervened in case of serious abnormal registrations (part-time). Access to registered data by the care provider was password-protected.

5-xi) Report any prompts/reminders used

Patients received feedback via e-mail and SMS (once weekly), encouraging them to gradually achieve predefined exercise training goals. In addition patients received e-mails and/or SMS's (once weekly) with tailored dietary and smoking cessation recommendations, based on cardiovascular risk factors at entry of study.

In case the patients did not send/upload their data; the system sent a reminder email/SMS (also on a weekly basis).

5-xii) Describe any co-interventions (incl. training/support)

Control patients received standard cardiac rehabilitation.
Intervention patients received standard cardiac rehabilitation plus the telerehabilitation intervention.

The standard cardiac rehabilitation included a 12-week center-based CR program, including 45 pluridisciplinary rehabilitation sessions with at least two exercise training sessions per week10. Patients were instructed to exercise for 45-60 min per session at a target heart rate and/or workload corresponding to an intensity between their first ventilatory threshold (VT1, as detected by V-slope method) and respiratory compensation point (RCP, as detected by carbon dioxide equivalent (VE/VCO2) slope method). Endurance training consisted of walking/running, and/or cycling and arm cranking.

Note: CR = cardiac rehabilitation

6a) CONSORT: Completely defined pre-specified primary and secondary outcome measures, including how and when they were assessed

The outcome measures were described in the methods section as follows:

"All outcome assessors were blinded to group allocation. The primary outcome measure was peak aerobic capacity (VO2peak); measured during maximal cardiopulmonary exercise testing 11 with breath-by-breath gas exchange analysis at baseline, after 6 and 24 weeks of study period. The CPET was maximal in case of an achieved heart rate >85% of the maximal predicted heart rate, a Respiratory gas Exchange Ratio (RER) >1.1, and/or a ventilatory reserve (VR: VE peak/MVV) >80%11. The first ventilatory threshold (VT1) and the oxygen uptake efficiency slope (OUES) were used as surrogate markers for VO2peak in case of submaximal CPET. VT1 was defined by the V-slope method, OUES was calculated using the method of Baba et al.12. Two independent investigators, blinded to treatment allocation interpreted CPET reports.
The first secondary outcome measure was daily physical activity13; both registered by triaxial accelerometry (Yorbody sensor) and self-assessed by the patient. The accelerometer provided daily recordings of aerobic (defined as sustained activity at ≥60 steps/min for ≥10 minutes), regular (activity at <60 steps/min) and total (sum of aerobic and regular) steps. Self-reported physical activity was based on the offline IPAQ questionnaire, completed at baseline, after 6 and 24 weeks. MET-minutes were computed by multiplying predefined MET-scores by the minutes of a specific activity performed, to weigh each type of activity by its energy requirement (for the domain leisure time activity and for all domains together). Following MET-scores were used: 3.3 METs for walking, 4.0 METs for moderate and 8.0 METs for vigorous physical activity, respectively.
The 14-item offline HeartQol questionnaire 14 was used to assess health-related quality of life (HRQL) at study start, after 6 and 24 weeks. Mean (± SD) scores were calculated for both the physical (10-item) and emotional (4-item) subscale. Cronbach's α evaluated internal consistency reliability for the global score and each subscale. The proportion of patients at the floor ("floor effect", defined as the lowest possible score on the questionnaire) and at the ceiling ("ceiling effect", defined as the best possible score) was determined to assess sensitivity to positive and negative changes in HRQL."

6a-i) Online questionnaires: describe if they were validated for online use and apply CHERRIES items to describe how the questionnaires were designed/deployed

Outcomes were obtained through offline (not online) questionnaires.

6a-ii) Describe whether and how "use" (including intensity of use/dosage) was defined/measured/monitored

Use was measured by data transmission and/or login registration. The average number of logins per patient per week was calculated.

6a-iii) Describe whether, how, and when qualitative feedback from participants was obtained

All intervention patients provided feedback through feedback forms. These forms were given to the intervention patients after study completion.

6b) CONSORT: Any changes to trial outcomes after the trial commenced, with reasons

There were no changes to trial outcomes after the trial commenced.

7a) CONSORT: How sample size was determined

7a-i) Describe whether and how expected attrition was taken into account when calculating the sample size

A priori sample size calculation yielded 140 necessary patients to detect a 20% effect size of the primary outcome measure (VO2 peak) between groups (IG vs CG); with a statistical power of 95% at a 2-sided type I error level of 0.05 and a dropout rate of 30%.

7b) CONSORT: When applicable, explanation of any interim analyses and stopping guidelines

Not applicable.

8a) CONSORT: Method used to generate the random allocation sequence

The patients were randomly assigned (1:1) to internet-based telerehabilitation in addition to center-based rehabilitation (intervention group (IG)) or center-based rehabilitation alone (control group (CG)). A central computerized randomization system, using block randomization, ascertained equal distribution of patients in the different recruiting hospitals for both treatment arms.

8b) CONSORT: Type of randomisation; details of any restriction (such as blocking and block size)

A central computerized randomization system, using block randomization, ascertained equal distribution of patients in the different recruiting hospitals for both treatment arms.

9) CONSORT: Mechanism used to implement the random allocation sequence (such as sequentially numbered containers), describing any steps taken to conceal the sequence until interventions were assigned

Since we used a computerized randomization system; there were no concealment problems.

10) CONSORT: Who generated the random allocation sequence, who enrolled participants, and who assigned participants to interventions

A central computerized randomization system generated the random allocation sequence and assigned participants to the interventions.

Patients were enrolled by the principal investigator of Telerehab III, when they met the eligibility criteria.

11a) CONSORT: Blinding - If done, who was blinded after assignment to interventions (for example, participants, care providers, those assessing outcomes) and how

11a-i) Specify who was blinded, and who wasn't

Given the nature of the intervention, it was not possible to blind the participants. However, the outcome assessors and those doing data analysis were blinded.

11a-ii) Discuss e.g., whether participants knew which intervention was the "intervention of interest" and which one was the "comparator"

Patients knew which intervention was the "intervention of interest" and which one was the "comparator". They were informed about both treatment arms prior to inclusion in the trial by an information session (lasting one hour).

11b) CONSORT: If relevant, description of the similarity of interventions

Not relevant.

12a) CONSORT: Statistical methods used to compare groups for primary and secondary outcomes

Data analysis was performed using SPSS v. 22 according to the intention-to-treat principle, by assigned treatment group. Nonparametric alternatives were used for parametric statistics in case assumptions for the latter were violated. The Shapiro-Wilk test was used to assess normality. Paired t-tests (parametric) or Wilcoxon signed-rank tests (non-parametric) were used for within-group analysis; independent t-tests (parametric) or Mann-Whitney U tests (non-parametric) for between-group analysis. Repeated measures ANOVA (parametric) or Friedman's ANOVA (non-parametric) compared multiple dependent means. Chi-square tests were used in case of categorical variables, Fisher's exact tests were used when expected frequencies were small. Pearson's (r) or Spearman's (rs) correlation coefficients were calculated to express relationships between variables (bivariate correlations). The significance level for tests was 2-sided α of 0.05. Effect sizes for the HeartQol questionnaire were reported using the standardized response mean (SRM) methodology [SRM = (A –B)/D], where A and B are the mean scores at time 2 and time 1, respectively. D represents the score change standard deviation. Sensitivity analysis of accelerometric activity measurements was performed to cope with incomplete activity registrations. Inclusion thresholds of 1,000, 2,000, 3,000, 4,000 and 5,000 total daily steps or ≥ 7, ≥ 8 and ≥ 9 daily measurement hours were arbitrarily chosen; because these represented reliable registrations. All available data were used, no data imputation was performed for missing values.

12a-i) Imputation techniques to deal with attrition / missing values

All available data were used, no data imputation was performed for missing values.

12b) CONSORT: Methods for additional analyses, such as subgroup analyses and adjusted analyses

There were no additional analyses, such as subgroup analyses and adjusted analyses in this Telerehab III trial.

RESULTS

13a) CONSORT: For each group, the numbers of participants who were randomly assigned, received intended treatment, and were analysed for the primary outcome

Telerehab III was a multi-center, prospective, randomized, controlled clinical trial, run at Jessa Hospital (Hasselt) (n = 103), Ziekenhuis-Oost Limburg (Genk) (n = 27) and St. Franciscus Hospital (Heusden-Zolder) (n = 10).

70 patients were randomly assigned to the intervention group. 70 patients received the intervention. 69 intervention patients were analysed for the primary outcome.

70 patients were randomly assigned to the control group. 70 patients received standard cardiac rehabilitation. 70 control patients were analysed for the primary outcome.

13b) CONSORT: For each group, losses and exclusions after randomisation, together with reasons

The flow diagram illustrating this information is part of the paper.

13b-i) Attrition diagram

The attrition diagram is included in the paper.

14a) CONSORT: Dates defining the periods of recruitment and follow-up

Telerehab III recruited patients between February 2013 and 2015.

The follow-up period per patient was 24 weeks.

14a-i) Indicate if critical "secular events" fell into the study period

Critical "secular events" did not fall into the study period

14b) CONSORT: Why the trial ended or was stopped (early)

The trial was not ended or stopped early.
The trial ended when all participants completed their 24-weeks trial period.

15) CONSORT: A table showing baseline demographic and clinical characteristics for each group

The requested table in included in the paper.

15-i) Report demographics associated with digital divide issues

Age and gender of the participants were included in the table.

Since computer/internet/ehealth literacy was an inclusion criterium; all participants were computer/internet/ehealth literate.

16a) CONSORT: For each group, number of participants (denominator) included in each analysis and whether the analysis was by original assigned groups

16-i) Report multiple "denominators" and provide definitions

This information is included in the paper.

16-ii) Primary analysis should be intent-to-treat

Data analysis was performed according to the intention-to-treat principle, by assigned treatment group.

17a) CONSORT: For each primary and secondary outcome, results for each group, and the estimated effect size and its precision (such as 95% confidence interval)

For aerobic capacity: Mean VO2 peak improved significantly in IG patients from baseline [22.46 ± 0.78 ml/min*kg] to 24 weeks [24.46 ± 1.00 ml/min*kg] ($p < 0.001$). In the CG mean VO2 peak did not change after 24 weeks when compared to baseline ($p = 0.089$), and decreased from week 6 [22.86 ± 0.66 ml/kg/min] to week 24 [22.15 ± 0.77 ml/min*kg] ($p = 0.018$), after an initial non-significant increase. Between-group analysis of aerobic capacity was significant after 24 weeks ($p < 0.001$) in favor for the IG.

For physical activity: Summed vigorous-moderate-walking activity (VMW) leisure increased significantly in the IG (χ^2 F (2) = 13.66, $p = 0.001$) during study period. In the CG VMW leisure (MET-min/week) did not change (χ^2 F (2) = 0.646, $p = 0.724$), however it showed a downward trend. Between-group analysis confirmed a difference between the IG and CG in favor of the IG (U = 1,830, z = 3.336, $p = 0.001$).

For quality of life: IG patients showed a significant improvement in perceived health-related quality of life (QoL) for the physical subscale from baseline (2.23 ± 0.08) to the end of study period (2.52 ± 0.07) (χ^2 F (2) = 15.35, $p < 0.001$). The SRM of 0.43 indicated a small to moderate effect size. Their global QoL score also improved significantly (χ^2 F (2) = 14.04, $p < 0.001$). The SRM of 0.43 indicated a small to moderate effect size. The QoL of the CG patients did not change during study period for the physical subscale (χ^2 F (2) = 6.32, $p = 0.05$), the emotional subscale (χ^2 F (2) = 0.456, $p = 0.80$) or the global scale (χ^2 F (2) = 3.11, $p = 0.21$). Between-group analysis confirmed that globally the IG's QoL improved more compared to the CG (U = 2,407, z = 2.805, $p = 0.005$).

The results were thus included in the paper. The precision of the data was given by means of including standard deviations (and not only mean values). Effect sizes were reported.

17a-i) Presentation of process outcomes such as metrics of use and intensity of use

The number and reasons for dropout patients is included in the paper.

Regarding metrics of use: Use was measured by data transmission and/or login registration. The average number of logins per patient per week was calculated. Intervention patients transmitted their activity data 1.0 ± 0.3 times per week.

17b) CONSORT: For binary outcomes, presentation of both absolute and relative effect sizes is recommended

Effect sizes were reported in the paper.

18) CONSORT: Results of any other analyses performed, including subgroup analyses and adjusted analyses, distinguishing pre-specified from exploratory

There were no other analyses performed (including subgroup analyses and adjusted analyses, distinguishing pre-specified from exploratory).

18-i) Subgroup analysis of comparing only users

There was no subgroup analysis of comparing only users.

19) CONSORT: All important harms or unintended effects in each group

There were no important harms or unintended effects reported in the paper, since there were no important harms or unintended effects reported in the Telerehab III trial.

19-i) Include privacy breaches, technical problems

Patients did not report privacy breaches and/or other unexpected/unintended incidents (both during and after study period).

Technical problems were registered by the independent person responsible for technical assistance in case of sensor/system failure. These data were not included in the final paper; however in general there was only one patient who reported a lot of technical problems (and for this reason dropped out of the study).

19-ii) Include qualitative feedback from participants or observations from staff/researchers

All intervention patients provided qualitative feedback through feedback forms. These forms were given to the intervention patients after study completion.

The majority of intervention patients liked the telerehabilitation program. The majority of patients was willing to use the accelerometer and/or webservice after study completion.

DISCUSSION

20) CONSORT: Trial limitations, addressing sources of potential bias, imprecision, multiplicity of analyses

20-i) Typical limitations in ehealth trials

Subitem 20-i is addressed in the discussion section as follows: "Potential bias of the results due to non-use of the tele-intervention was not excluded."

21) CONSORT: Generalisability (external validity, applicability) of the trial findings

21-i) Generalizability to other populations

Subitem 21-i was addressed in te discussion section as follows: "A limitation of this study was that Telerehab III was initially designed to recruit a broad cardiac patient population (including both CAD, CHF with reduced EF and CHF with preserved EF). However, as shown by the baseline clinical characteristics (Table 1), only a minority of CHF patients eventually participated (5.8 % and 7.1 % in the IG and CG respectively). This reduced the generalizability of study findings to CHF patients. "

21-ii) Discuss if there were elements in the RCT that would be different in a routine application setting

Subitem 21-ii was addressed in the discussion section as follows: "Finally, in Telerehab III one part-time (caregiver) was responsible for control of feedback content and one part-time (technical assistant) for system/service operability. In a routine application setting similar staff requirements would be sufficient."

22) CONSORT: Interpretation consistent with results, balancing benefits and harms, and considering other relevant evidence

22-i) Restate study questions and summarize the answers suggested by the data, starting with primary outcomes and process outcomes (use)

Subitem 22-i is addressed in the discussion section as follows:
"The present study showed that the addition of a patient-specific, comprehensive telerehabilitation program to conventional center-based CR can improve both physical fitness and associated health-related quality of life at 6 months follow-up; compared to center-based CR alone. Peak oxygen uptake (VO2peak (ml/min*kg)), daily total step count and IPAQ's self-reported VMW activities (MET-min/week) increased from baseline to 6 weeks in both treatment groups. They additionally increased between week 6 and 24 in the IG, but decreased in the CG. CG patients participated in the center-based CR during the first 6 weeks of study period only, probably explaining their initial improvement in outcome measures. The observed findings imply that CG patients return to prior lifestyle behavior after center-based CR, while IG patients further maintain and ameliorate acquired behavioral change."

22-ii) Highlight unanswered new questions, suggest future research

The new question that is unanswered is whether the findings of Telerehab III persist for a longer period than the period assessed.

This item is addressed in the paper as follows: "We plan to conduct a follow-up trial of Telerehab III (starting in August 2015) to assess whether the intervention-related health benefits persist 2 years after study termination. "

Other information

23) CONSORT: Registration number and name of trial registry

The study protocol was registered at the ISRCTN registry with registration number ISRCTN29243064.

24) CONSORT: Where the full trial protocol can be accessed, if available

The full trial protocol can be accessed at: Frederix I, Van Craenenbroeck EM, Vrints C, Dendale et al. Telerehab III: a multi-center randomized, controlled trial investigating the long-term effectiveness of a comprehensive cardiac telerehabilitation program - Rationale and study design. BMC Cardiovasc Disord 2015;15(1):29.

25) CONSORT: Sources of funding and other support (such as supply of drugs), role of funders

The Telerehab III trial was funded by a research grant from Flanders Care [grant number DEM2012-02-03] and from the Research Foundation Flanders (FWO) [grant number: 1128915N]. The authors/evaluators of the Telerehab III intervention are distinct from the intervention developers.

X26-i) Comment on ethics committee approval

The study was conducted in accordance with the principles stated in the Declaration of Helsinki (reviewed version of 2008), local and national regulations. The study protocol was approved by Jessa Ethics Committee (reference number: B243201216043).

x26-ii) Outline informed consent procedures

All patients provided offline informed consent, including a statement on the content of the intervention of interest, prior to study enrollment.

The content of the informed consent was based on the federal agency for medicines and health products (famhp) templates (reference: http://www.fagg-afmps.be/en/human_use/medicines/medicines/research_development/ethic_committee/templates_informed_consent/).

X26-iii) Safety and security procedures

The Telerehab III team ensured that patients' personal data that derived from this trial, were treated confidentially and anonymously conform "Law on patient personal data protection and privacy related to treatment (08/12/1992)". (Reference: http://www.ejustice.just.fgov.be/cgi_loi/change_lg.pl?language=nl&la=N&table_name=wet&cn=1992120832)

X27-i) State the relation of the study team towards the system being evaluated

The author(s) declare that they have no conflict of interest.

The authors/evaluators of the Telerehab III intervention are distinct from the intervention developers.
