# Supplementary material for: Medium-Term Effectiveness of a Comprehensive Internet-Based and Patient-Specific Telerehabilitation Program With Text Messaging Support for Cardiac Patients: Randomized Controlled Trial
Source: J Med Internet Res. 2015 Jul 23;17(7):e185. doi: 10.2196/jmir.4799 (PMC4528085; doi:10.2196/jmir.4799)

Multimedia appendix 3. Screenshot of the website with an example SMS sent to the patient.

The webpage shows the patient's physical activity level. An example SMS is shown on the right side of the picture, underneath it you can see the Yorbody accelerometer. For simplicity, only one of the screenshots is included in the appendix. The patient "Jan" was a pilot test person, not actually a real patient (for privacy of personal sensitive data reasons).

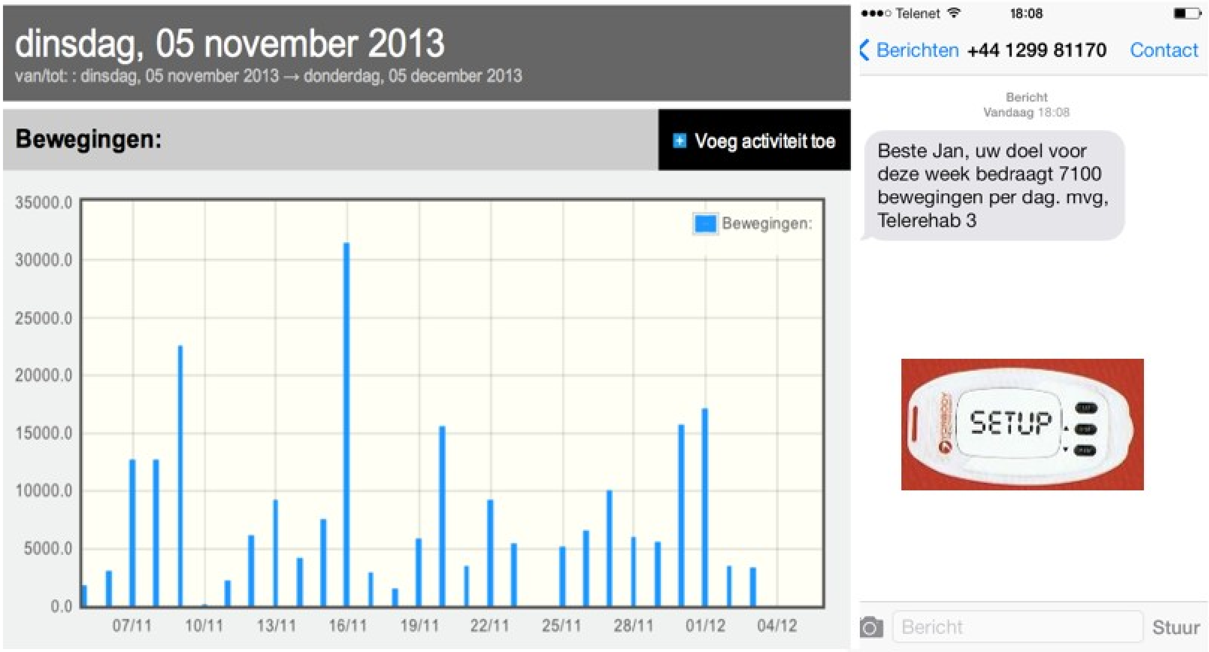

Supplement: Multimedia Appendix 3 [file jmir_v17i7e185_app3.pdf]
