# Supplementary material for: Medium-Term Effectiveness of a Comprehensive Internet-Based and Patient-Specific Telerehabilitation Program With Text Messaging Support for Cardiac Patients: Randomized Controlled Trial
Source: J Med Internet Res. 2015 Jul 23;17(7):e185. doi: 10.2196/jmir.4799 (PMC4528085; doi:10.2196/jmir.4799)
Supplement: Multimedia Appendix 4 [file jmir_v17i7e185_app4.pdf]

Multimedia appendix 4. Feedback form for Telerehab III intervention group.

1. Was it difficult to combine the prescribed physical activities with your daily life? Yes/No
2. Do you think your physical activities contribute to better health? Yes/No
3. Was the accelerometer easy to read? Yes/No
4. Was it sufficient for the accelerometer to measure only walking and/or running activities? Yes/No
5. How much time (minutes) did you spend on physical exercise prior to study start? …….. minutes
6. Did you feel safer by using the accelerometer in combination with the telemonitoring and telecoaching service? Yes/No
7. Did you gain more insight in your health condition by being included in the Telerehab III program? Yes/No
8. Did the Telerehab III intervention prompt you to increase your daily activity level? Yes/No
9. Do you prefer A) telecoaching via the accelerometer B) telecoahing via email or C) telecoaching via SMS? A/B/C
10. Did you find the Telerehab III intervention interfering with your daily activities? Yes/No
11. Was the accelerometer easy to use? Yes/No
12. Did you sometimes forget to wear the accelerometer? Yes/No
13. Did you feel fitter by using the Telerehab III intervention? Yes/No
14. Was it easy to comply with the prescribed exercises? Yes/No
15. Are you more reassured, knowing you are monitored by the Telerehab system? Yes/No
16. Would you like to continue using the Telerehab III service after study completion? Yes/No
17. How much time (minutes) do you spend on physical exercise since study start?...... minutes

18. Do you think the Telerehab III program is privacy intrusive? Yes/No

19. Did you have difficulties uploading physical activity data to the webpage? Yes/No

20. Do you consider the fact that the accelerometer can only register walking/running activities a limitation of the sensor? Yes/No

21. Would you like the sensor to be able to monitor other activities than walking and/or running? Yes/No

22. What is your general opinion about the Telerehab III system? I am very satisfied/satisfied/no opinion/not satisfied.
