# Supplementary material for: Medium-Term Effectiveness of a Comprehensive Internet-Based and Patient-Specific Telerehabilitation Program With Text Messaging Support for Cardiac Patients: Randomized Controlled Trial
Source: J Med Internet Res. 2015 Jul 23;17(7):e185. doi: 10.2196/jmir.4799 (PMC4528085; doi:10.2196/jmir.4799)
Supplement: Multimedia Appendix 5 [file jmir_v17i7e185_app5.pdf]

Multimedia appendix 5. Complete set of measured CPET parameters at baseline, 6 weeks and 24 weeks follow-up period.

				Within-group				Between-group			
			Week 24	*P*	*P*	*P*	*P*	*P*	*P*	*P*	*P*
**Intervention (all RER)**	***Mean (±SD)***	***Mean (±SD)***	***Mean (±SD)***	***Overall***	***Δ6-1***	***Δ24-6***	***Δ24-1***	***Overall***	***Δ6-1***	***Δ24-6***	***Δ24-1***
RERmax	1.15 (± 0.10)	1.15 (± 0.10)	1.18 (± 0.12)	.01	.99	.02	.18	.38	NA	NA	NA
Gewicht (kg)	83.3 (± 18.2)	83.2 (± 17.4)	83.0 (± 17.3)	.69	NA	NA	NA	.45	NA	NA	NA
BMI (kg/m2)	28 (± 5)	28 (± 5)	28 (± 5)	.63	NA	NA	NA	.60	NA	NA	NA
HR rest (beats/min)	73 (± 14)	72 (± 12)	70 (± 13)	.38	NA	NA	NA	.88	NA	NA	NA
HR max (beats/min)	127 (± 22)	130 (± 20)	132 (± 20)	.11	NA	NA	NA	.71	NA	NA	NA
DBP rest (mmHg)	82 (± 19)	81 (± 21)	77.24 (± 21.13)	.48	NA	NA	NA	.67	NA	NA	NA
DBP max (mmHg)	94 (± 27)	94 (± 22)	84 (± 24)	.23	NA	NA	NA	.73	NA	NA	NA
SBP rest (mmHg)	126 (± 21)	129 (± 30)	150 (± 140)	.26	NA	NA	NA	.30	NA	NA	NA
SBP max (mmHg)	168 (± 31)	177 (± 32)	172 (± 38)	.15	NA	NA	NA	.05	.01	.77	.04
RPP rest (mmHg*beats/min)	9,170 (± 2480)	9,188 (± 2451)	10,663 (± 11526)	.30	NA	NA	NA	.31	NA	NA	NA
RPP max (mmHg*beats/min)	21,677 (± 6554)	23,138 (± 5794)	22,985 (±6856)	.19	NA	NA	NA	.37	NA	NA	NA
VO2 peak (ml/min*kg)	22.46 (± 6.43)	23.91 (± 6.74)	24.46 (± 7.57)	.01	.08	.38	.01	<.001	.19	.01	<.001
HR max (%pred)	79 (± 13)	80 (± 12)	83 (± 12)	.047	.99	.36	.05	.53	NA	NA	NA
Watt (W)	152 (± 48)	163 (± 52)	165 (± 53)	.01	.02	.81	.01	.01	.90	.01	.02
Watt (pred%)	103 (± 23)	110 (± 27)	116 (± 27)	<.001	.01	.27	<.001	<.001	.83	.01	.01
VT1 (W)	69 (± 24)	75 (± 25)	81 (± 26)	<.001	.74	<.001	<.001	<.001	.80	<.001	<.001
VT1 (bpm)	93 (± 17)	91 (± 15)	96 (± 15)	.01	.99	.01	.08	.01	.35	.01	.047
OUES (ml/min/log(ml/min))	2,067 (± 518)	2,241 (± 545)	2,272 (± 579)	<.001	.02	.045	<.001	.1	NA	NA	NA
**Control (all RER)**											
RERmax	1.13 (± 0.08)	1.14 (± 0.09)	1.16 (± 0.11)	.01	.60	.01	.01				
Gewicht (kg)	82.7 (± 13.4)	82.5 (± 13.3)	82.5 (± 13.9)	.18	NA	NA	NA				
BMI (kg/m2)	28 (± 4)	28 (± 4)	27 (± 5)	.51	NA	NA	NA				
HR rest (beats/min)	73 (± 12)	73 (± 12)	72 (± 13)	.70	NA	NA	NA				
HR max (beats/min)	121 (± 24)	125 (± 21)	127 (± 22)	.12	NA	NA	NA				
DBP rest (mmHg)	84 (± 21)	78 (± 19)	79 (± 17)	.33	NA	NA	NA				
DBP max (mmHg)	94 (± 30)	94 (± 31)	86 (± 20)	.59	NA	NA	NA				
SBP rest (mmHg)	129 (± 25)	127 (± 23)	129 (± 21)	.57	NA	NA	NA				
SBP max (mmHg)	175 (± 30)	172 (± 34)	171 (± 31)	.26	NA	NA	NA				
RPP rest (mmHg*beats/min)	9,379 (± 2674)	9,273 (± 2703)	9,269 (± 2246)	.95	NA	NA	NA				
RPP max (mmHg*beats/min)	21,390 (± 6325)	22,061 (± 6254)	21,828 (± 5509)	.99	NA	NA	NA				
VO2 peak (ml/min*kg)	22.72 (± 6.05)	22.86 (± 5.37)	22.15 (± 5.83)	.02	.99	.02	.09				
HR max (%pred)	77 (± 12)	79 (± 13)	79 (± 12)	.43	NA	NA	NA				
Watt (W)	150 (± 49)	158 (± 50)	152 (± 53)	.01	<.001	.02	.99				
Watt (pred%)	105 (± 26)	108 (± 26)	104 (± 27)	.01	<.001	.01	.99				
VT1 (W)	83 (± 34)	88 (± 34)	76 (± 31)	<.001	.20	<.001	.01				
VT1 (bpm)	95 (± 15.	96 (± 15)	95 (± 17)	.53	NA	NA	NA				
OUES (ml/min/log(ml/min))	2,493 (± 2338)	2,263 (± 636)	2,142 (± 635)	.25	NA	NA	NA				
