# Supplementary material for: Medium-Term Effectiveness of a Comprehensive Internet-Based and Patient-Specific Telerehabilitation Program With Text Messaging Support for Cardiac Patients: Randomized Controlled Trial
Source: J Med Internet Res. 2015 Jul 23;17(7):e185. doi: 10.2196/jmir.4799 (PMC4528085; doi:10.2196/jmir.4799)

Multimedia appendix 6.

A Sensitivity analysis step data.

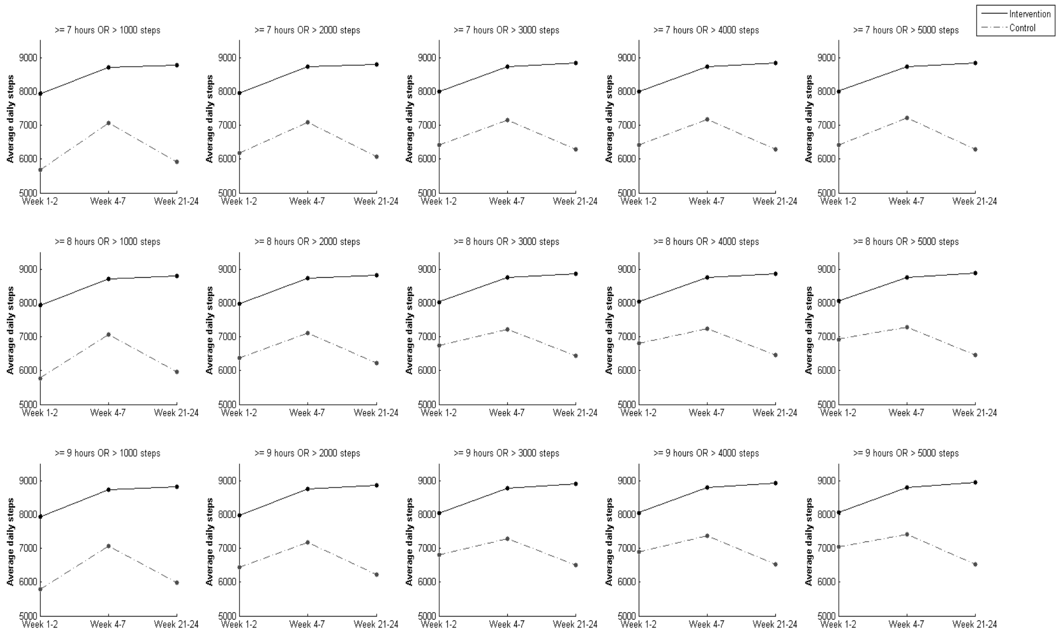

B Use of telemonitoring system.

This Figure shows the % intervention patients with > 2000 total daily steps or ≥ 8 daily measurement hours averaged per week.

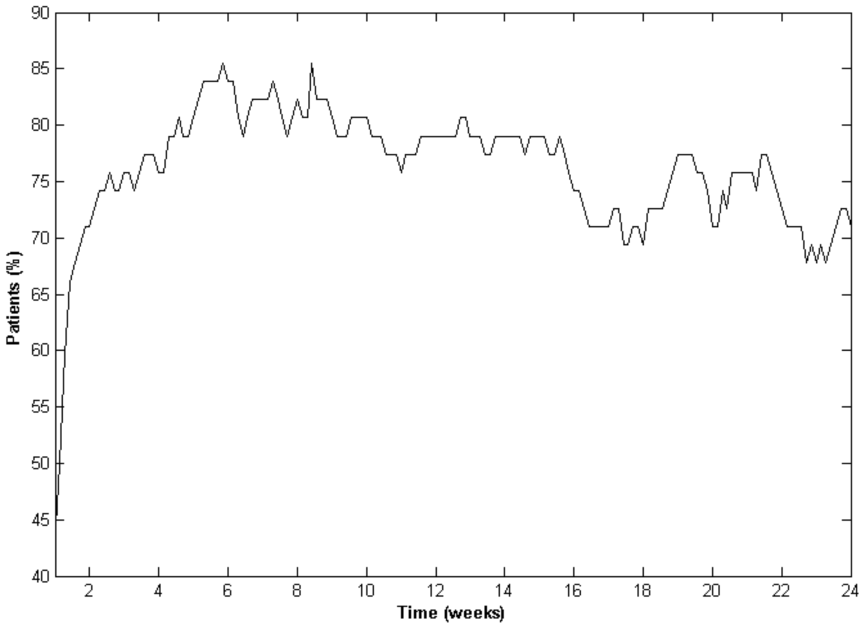

Supplement: Multimedia Appendix 6 [file jmir_v17i7e185_app6.pdf]
