# Supplementary material for: Medium-Term Effectiveness of a Comprehensive Internet-Based and Patient-Specific Telerehabilitation Program With Text Messaging Support for Cardiac Patients: Randomized Controlled Trial
Source: J Med Internet Res. 2015 Jul 23;17(7):e185. doi: 10.2196/jmir.4799 (PMC4528085; doi:10.2196/jmir.4799)
Supplement: Multimedia Appendix 7 [file jmir_v17i7e185_app7.pdf]

Multimedia appendix 7.

IPAQ	Intervention group		Control group	
**V (MET-min/week)**	***Mean (±SD)***	***Median [IQR]***	***Mean (±SD)***	***Median [IQR]***
Baseline	302 (± 729)	0 [0]	369 (± 827)	0 [240]
Week 6	465 (± 1231)	0 [360]	330 (± 1,086)	0 [0]
Week 24	648 (± 1953)	0 [320]	190 (± 537)	0 [0]
**M (MET-min/week)**				
Baseline	362 (± 703)	0 [420]	592 (± 1,084)	240 [720]
Week 6	603 (± 1110)	160 [720]	441 (± 518)	260 [720]
Week 24	619 (± 1049)	240 [720]	405 (± 757)	0 [720]
**W (MET-min/week)**				
Baseline	398 (± 573)	198 [446]	634 (± 1,220)	281 [718]
Week 6	625 (± 705)	297 [1,089]	543 (± 1,297)	231 [594]
Week 24	796 (± 967)	462 [949]	491 (± 583)	231 [589]
**VMW (MET-min/week)**				
Baseline	1,101 (± 1,197)	594 [1,484]	1,563 (± 1,885)	1,017 [1,855]
Week 6	1,575 (± 1,479)	1,386 [1,871]	1,308 (± 1,941)	679 [1,654]
Week 24	2,077 (± 2,357)	1,371 [2,198]	1,075 (± 1,203)	627 [1,181]
**S(min/week)**				
Baseline	2,596 (± 1,665)	2,340 [1,350]	2,710 (± 1,911)	2,400 [1,853]
Week 6	2,320 (± 1,167)	2,220 [1,620]	2,329 (± 1,503)	2,100 [1,605]
Week 24	2,028 (± 929)	1,800 [1,140]	2,741 (± 1,833)	2,220 [1,680]

Results from IPAQ questionnaire at baseline, 6 weeks and 24 weeks of follow-up period.

SD: Standard deviation; IQR: interquartile range; V: vigorous activities; M: moderate activities; W: walking; VMW: vigorous, moderate and walking; S: sitting. Note: only leisure time domain is included for V, M and W.
